# Strain-based fatigue data for Ti–6Al–4V ELI under fully-reversed and mean strain loads

**DOI:** 10.1016/j.dib.2016.02.014

**Published:** 2016-02-09

**Authors:** Patricio E. Carrion, Nima Shamsaei

**Affiliations:** a Department of Mechanical Engineering, Mississippi State University, Box 9552, Mississippi State, MS 39762, USA; b Center for Advanced Vehicular Systems (CAVS), Mississippi State University, Box 5405, Mississippi State, MS 39762, USA

**Keywords:** Fatigue, Cyclic deformation, Mean stress effects, Titanium alloys, Strain-life experiments

## Abstract

This article presents the experimental data supporting the study to obtain the mean strain/stress effects on the fatigue behavior of Ti–6Al–4V ELI. A series of strain-controlled fatigue experiments on Ti–6Al–4V ELI were performed at four strain ratios (−1, −0.5, 0, and 0.5). Two types of data are included for each specimen. These are the hysteresis stress–strain responses for the cycle in a log_10_ increment, and the maximum and minimum stress–strain responses for each cycle. Fatigue lives are also reported for all the experiments.

Specifications Table

**Table:** 

Subject area	Engineering
More specific subject area	Fatigue of Metals
Type of data	Table (Microsoft Excel file format)
How data was acquired	Strain-controlled fatigue experiments (laboratory)
Data format	Raw and analyzed
Experimental factors	Fatigue specimens were machined from a wrought Ti–6Al–4V ELI bar that was annealed for 1 h at 1300 °F. The round specimens with reduced uniform gage section were further polished to 4000 FEPA surface finish. M-coat D was used as a protective coating on the gage section to prevent extensometer blades from causing any damage on the specimen during testing.
Experimental features	Strain-controlled fatigue tests were performed according to ASTM E606-04 [1]. Test Frequencies were adjusted to eliminate any temperature and strain rate effects. All test were conducted at room temperature with an average relative humidity of 41%.
Data source location	Center for Advanced Vehicular Systems (CAVS), Mississippi State University, Mississippi State, MS, USA
Data accessibility	Data is within this article.

## Value of the data

• The experimental data presented in this article can be used to provide basis for the cyclic deformation and fatigue behavior of Ti–6Al–4V ELI, a widely used material in biomedical and aerospace applications, under zero and non-zero mean strain/stress conditions.
• Most of the generated data on fatigue behavior of Ti–6Al–4V are related to high-cycle fatigue with the use of a stress-life approach. However, the presented data were collected using the strain-life approach, which has been proven to correlate low-cycle fatigue data in a better manner than stress-life [2]. The data in this article can be used to improve current fatigue models and elucidate the material’s behavior under strain-controlled cyclic loadings in presence of mean stresses.
• The presented data can be used as a benchmark for fatigue research of Ti–6Al–4V ELI under more complex cyclic loads.

## 1 Data

The data included in this paper were obtained from the strain-controlled fatigue tests on Ti–6Al–4V ELI. Mean strain/stress effects were studied by performing fatigue experiments at different strain ratios, *R*_ε_, and various strain amplitudes, *ε*_a_. For each specimen, two types of data, the hysteresis stress–strain responses and the peak (maximum)/valley (minimum) stress–strain responses, are available. The hysteresis stress–strain responses were collected in a log_10_ increment, while the peak/valley stress–strain responses were recorded at each cycle. The corresponding fatigue lives are also reported for all the experiments. All the data has been deposited to the Data in Brief (DiB) Dataverse: 10.7910/DVN/EXS3F5.

## 2 Experimental design, materials and methods

**Fig. 1:**
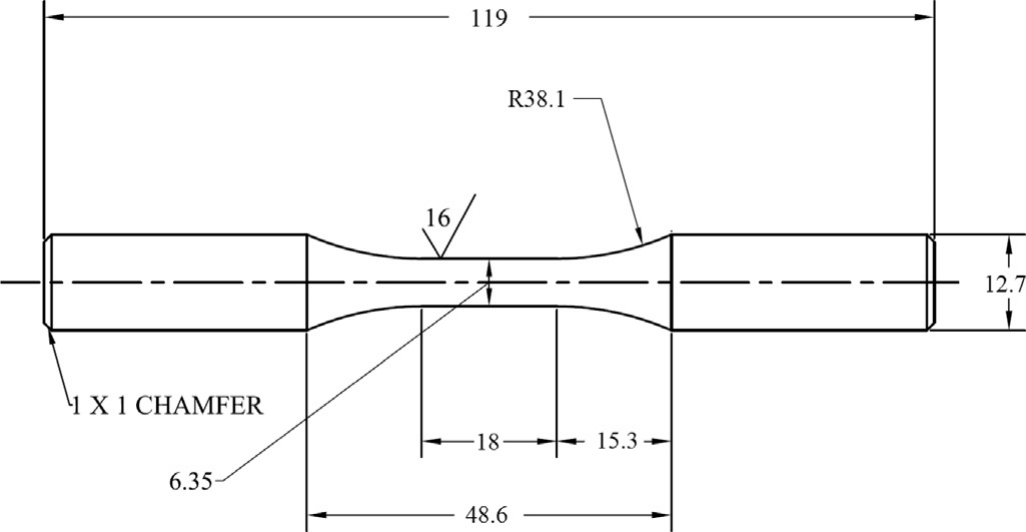
Fatigue specimen size and dimensions per ASTM standard E606/E606M-12 [1]. Dimensions presented in mm.

Fatigue specimens were machined from Ti–6Al–4V ELI Grade 5 round bar with 12.7 mm diameter to create round-shaped specimens with a reduced uniform gage section. The geometry and dimensions of the specimens, as illustrated in Fig. 1, were designed to comply with ASTM standard E606/E606M−12 [1]. Fatigue tests were performed at four strain ratios, including *R*_ε_=−1 (fully-reversed), *R*_ε_=−0.5 (tension–compression), *R*_ε_=0 (tension-release), and *R*_ε_=0.5 (tension–tension). For each strain amplitude, a minimum of two fatigue experiments were conducted to ensure that the test data was consistent. The strain amplitudes, *ε*_a_, ranged from 0.0015 to 0.012 mm/mm depending on the applied *R*_ε_. All tests were conducted at room temperature with an average 41% relative humidity, and using a servohydraulic test machine with a sinusoidal waveform input. The test frequency was adjusted for each strain amplitude to eliminate any temperature and strain rate effects on the cyclic behavior. Experiments that reached over 10^6^ cycles were determined to be a run-out and no duplicate test was performed. For some long life tests in the fully elastic region where the cyclic stress response was constant, the control mode was switched to load-control and the test frequency was increased to reduce the testing time. Table 1 summarizes the compiled data information for all strain-controlled fatigue tests, which were organized by the strain ratio, *R*_ε_, and the strain amplitude, *ε*_a_.

## Figures and Tables

**Table 1:** Ti-6Al-4 V ELI summary of strain-controlled fatigue tests.

**Specimen ID**	***R***_ε_	***ε***_***a***_	**2*****N***_***f***_
	**(mm/mm)**	**(Reversal)**
**Fully-Reversed Tests**			
sa 0.012 (1)	−1	0.012	2202
sa 0.012 (4)	−1	0.012	2336
sa 0.012 (5)	−1	0.012	3164
sa 0.010 (2)	−1	0.010	3986
sa 0.010 (4)	−1	0.010	4540
sa 0.010 (5)	−1	0.010	3760
sa 0.008 (1)	−1	0.008	14,338
sa 0.008 (2)	−1	0.008	12,914
sa 0.007 (3)	−1	0.007	49,812
sa 0.007 (4)	−1	0.007	49,174
sa 0.006 (3)	−1	0.006	124,952
sa 0.006 (5)	−1	0.006	207,610
sa 0.006 (6)	−1	0.006	128,050
sa 0.005 (1)	−1	0.005	>2,713,156
sa 0.004 (1)	−1	0.004	>4,431,694
sa 0.004 (2)	−1	0.004	>2,337,270
**Mean Strain Tests**			
sa 0.010 (1)R0	0	0.010	5464
sa 0.010 (2)R0	0	0.010	6724
sa 0.008 (1)R0	0	0.008	13,992
sa 0.008 (2)R0	0	0.008	11,398
sa 0.006 (1)R0	0	0.006	57,292
sa 0.006 (2)R0	0	0.006	65,472
sa 0.004 (1) R0	0	0.004	>2,506,668
sa 0.009 (1)R−0.5	−0.5	0.0090	11,546
sa 0.009 (2)R−0.5	−0.5	0.0090	10,516
sa 0.0075 (1)R−0.5	−0.5	0.0075	22,700
sa 0.0075 (2)R−0.5	−0.5	0.0075	22,992
sa 0.006 (3)R−0.5	−0.5	0.0060	128,248
sa 0.0045 (1)R-0.5	−0.5	0.0045	>2,371,298
sa 0.0045 (4)R0.5	0.5	0.0045	64,066
sa 0.0045 (6)R0.5	0.5	0.0045	98,788
sa 0.004 (3)R0.5	0.5	0.0040	230,884
sa 0.004 (4)R0.5	0.5	0.0040	129,922
sa 0.003 (2)R0.5	0.5	0.0030	>2,370,558
sa 0.003 (3)R0.5	0.5	0.0030	>2,104,496
sa 0.0025 (4)R0.5	0.5	0.0025	>2,319,500
sa 0.0020 (1)R0.5	0.5	0.0020	>2,047,510
sa 0.0015 (1)R0.5	0.5	0.0015	>2,048,000
